# Supplementary material for: Observing one-divalent-metal-ion dependent and histidine-promoted His-Me family I-PpoI nuclease catalysis in crystallo
Source: bioRxiv. 2024 May 5:2024.05.02.592236. Preprint. [Version 1] doi: 10.1101/2024.05.02.592236 (PMC11092635; doi:10.1101/2024.05.02.592236)
Supplement: Supplementary file 1 [file NIHPP2024.05.02.592236v1-supplement-1.pdf]

bioRxiv preprint doi: https://doi.org/10.1101/2024.05.02.592236; this version posted May 5, 2024. The copyright holder for this preprint (which was not certified by peer review) is the author/funder, who has granted bioRxiv a license to display the preprint in perpetuity. It is made available under aCC-BY 4.0 International license.

Supplementary Materials

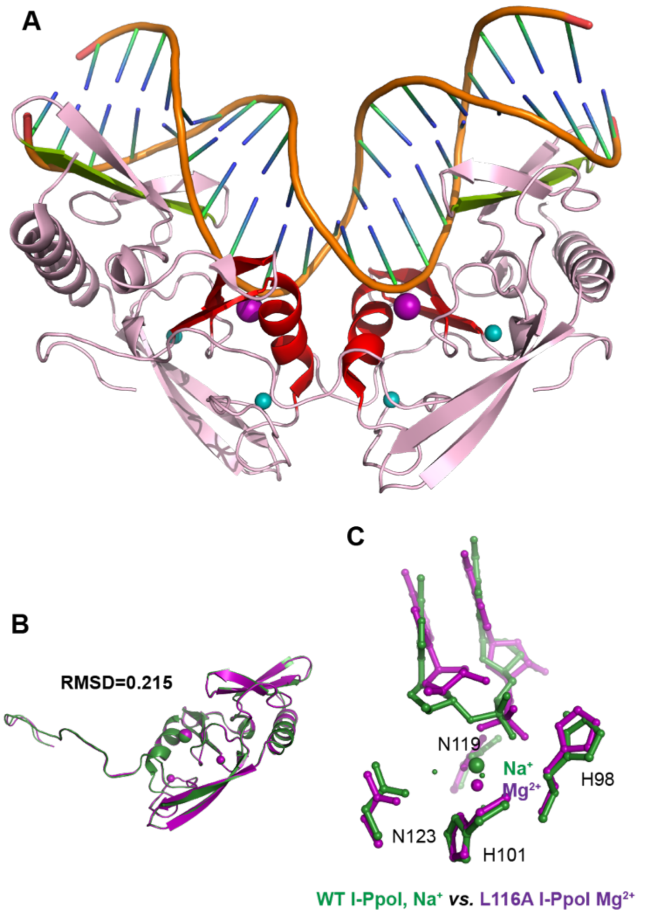

Fig. S1. Overall structure and catalytic core of homing endonuclease I-PpoI.
(**A**) Homing endonuclease I-PpoI (PDB ID 1CZO) binding as a dimer to bend DNA at 55˚. The overall endonuclease is colored in pink while the DNA is colored in orange. The catalytic core comprised of an alpha helix and two beta sheets are highlighted in red. The metal ion binding site is depicted as a purple sphere while the Zn^{2+} binding sites are depicted as turquoise spheres. The beta sheets involved in DNA binding are depicted in light green. (**B**) Monomer of homing endonuclease I-PpoI (PDB ID 1CZO) (green) superimposed on top of the other I-PpoI monomer (purple) within the same unit cell, resulting in a RMSD of 0.215. **c**, Structural comparison of the active site of WT I-PpoI (PDB ID 1CZ0) versus Leu116Ala I-PpoI (PDB ID 1EVW). The DNA fails to dock tightly towards the Mg^{2+} in the active site of Leu116Ala I-PpoI.

bioRxiv preprint doi: https://doi.org/10.1101/2024.05.02.592236; this version posted May 5, 2024. The copyright holder for this preprint (which was not certified by peer review) is the author/funder, who has granted bioRxiv a license to display the preprint in perpetuity. It is made available under aCC-BY 4.0 International license.

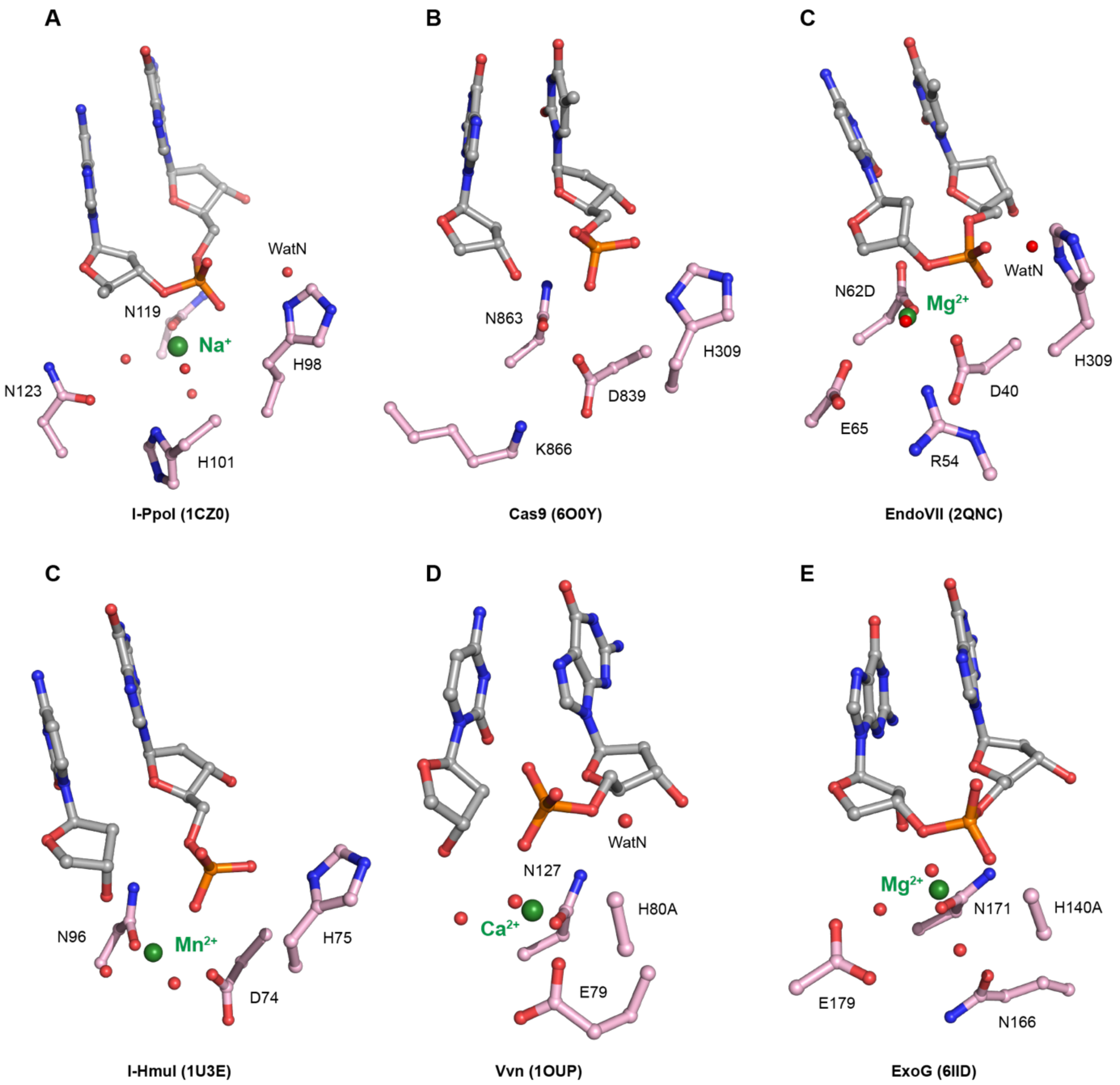

Fig. S2. Active sites of His-Me superfamily nucleases.
Carbon atoms of residues within the active site are colored in pink. The metal ions are depicted by green spheres while waters are depicted by red spheres.

bioRxiv preprint doi: https://doi.org/10.1101/2024.05.02.592236; this version posted May 5, 2024. The copyright holder for this preprint (which was not certified by peer review) is the author/funder, who has granted bioRxiv a license to display the preprint in perpetuity. It is made available under aCC-BY 4.0 International license.

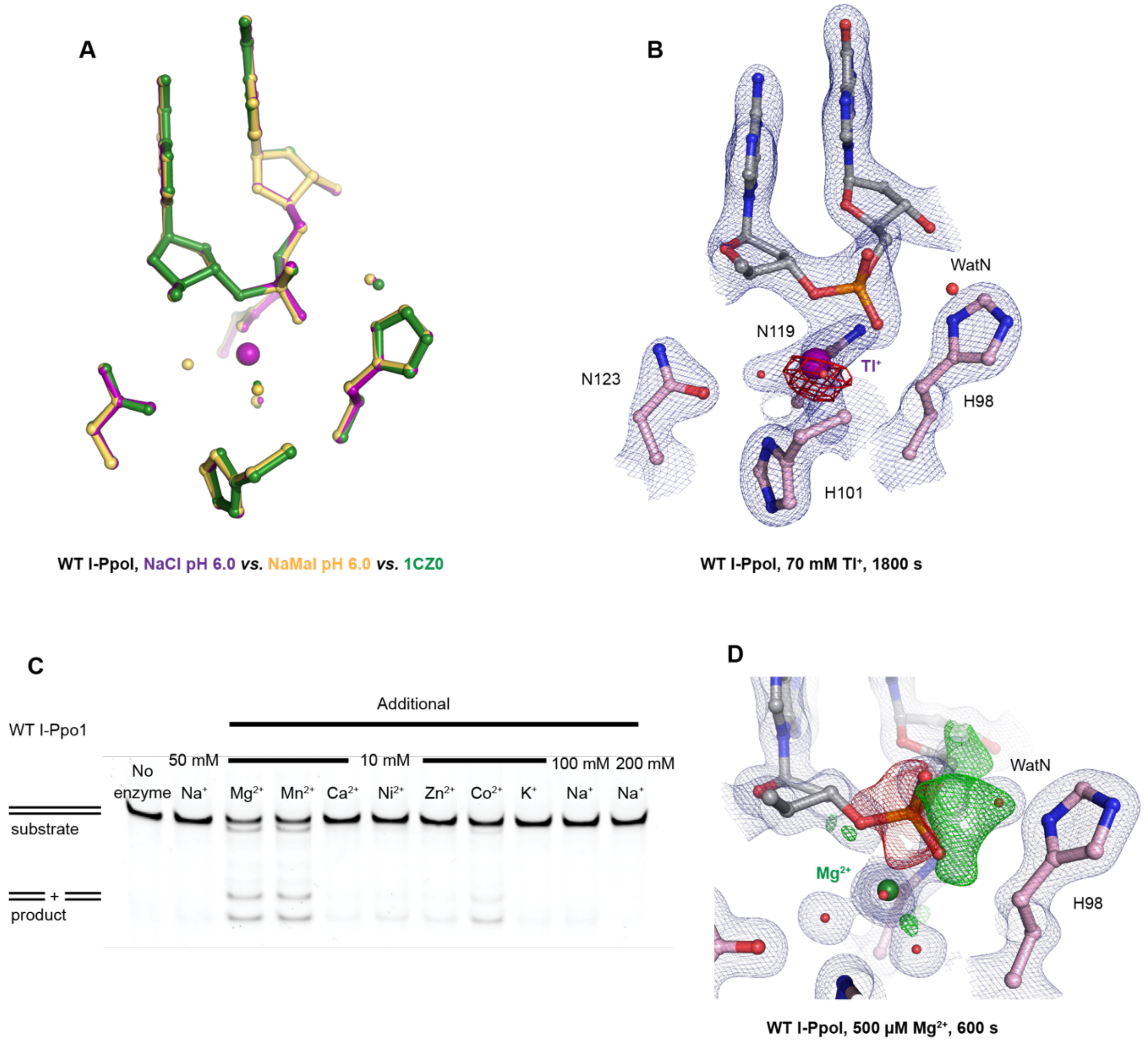

Fig. S3. Establishing I-PpoI for *in crystallo* studies.
(**A**) Structural comparison of the active site after soaking in NaCl in purple, sodium malonate in yellow and PDB ID 1CZ0 in green. (**B**) Tl^+ anomalous signal was detected at the I-PpoI metal ion binding site after 1800 s soaking in 70 mM Tl^+. The $2F_o$-F_c map for Me^{2+}, DNA, waters (red spheres), and catalytic residues (blue) was contoured at 2.0 σ. The anomalous map for Tl^+ was contoured at 3.0 σ. (**C**) Metal ion assay of 10 mM additional metal ions on I-PpoI DNA hydrolysis. (**D**) Negative F_o-F_c peaks (red) were detected on the leaving group side of the scissile phosphate while positive F_o-F_c peaks (green) were detected on the nucleophile side after 600 s Mg^{2+} soaking. The $2F_o$-F_c map for Me^{2+}, DNA, waters (red spheres), and catalytic residues (blue) was contoured at 2.0 σ. The negative F_o-F_c map for the reactant phosphate (red) and the positive F_o-F_c map for the product phosphate (green) were contoured at 3.0 σ.

bioRxiv preprint doi: https://doi.org/10.1101/2024.05.02.592236; this version posted May 5, 2024. The copyright holder for this preprint (which was not certified by peer review) is the author/funder, who has granted bioRxiv a license to display the preprint in perpetuity. It is made available under aCC-BY 4.0 International license.

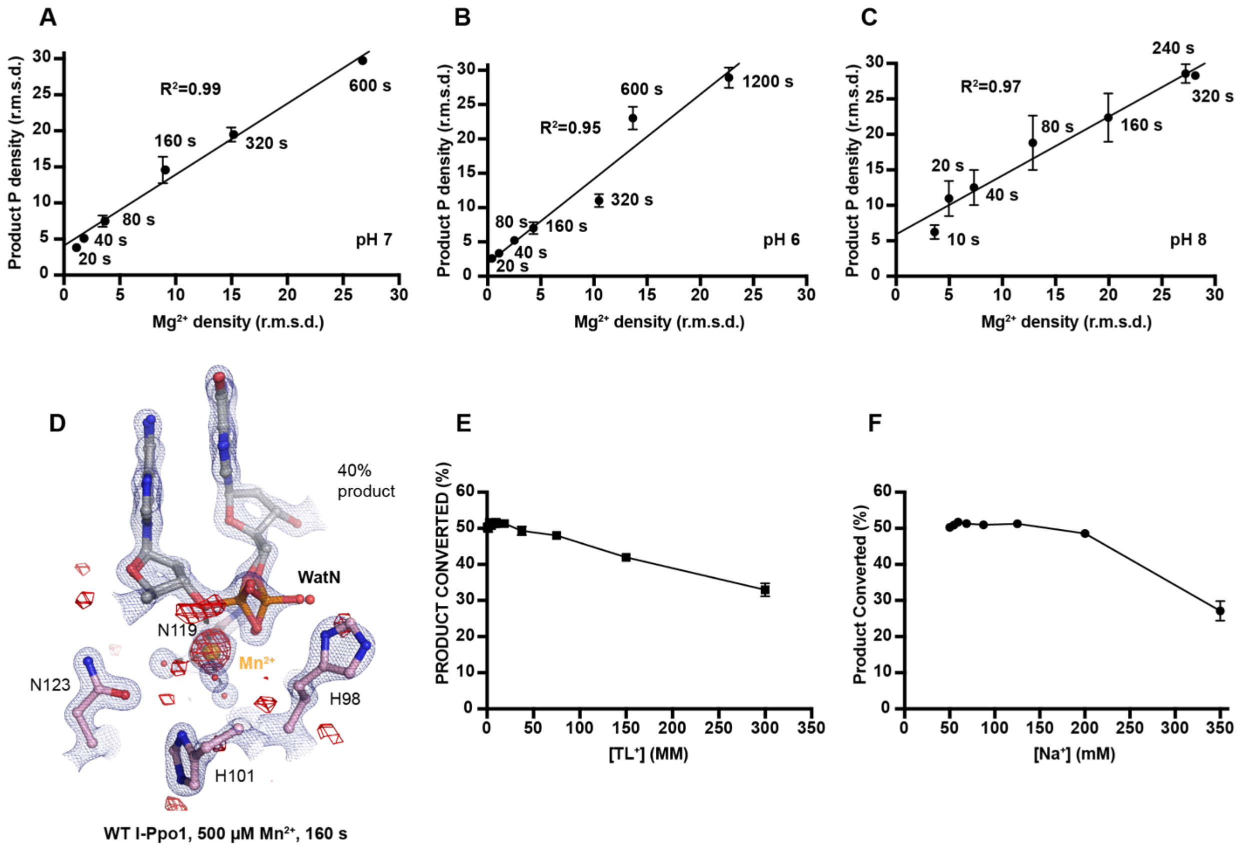

Fig. S4. Additional metal ions are not required for DNA hydrolysis by I-PpoI.
Correlation (R^2) between the newly formed phosphate and Mg^{2+} binding at pH 7 in (**A**) pH 6 in (**B**) and pH 8 in (**C**). (**A-C**) The points represent the mean of duplicate measurements for the electron density of the reaction product phosphate within two I-PpoI molecules in the asymmetric unit while the errors bars represent the standard deviation. (**D**) Mn^{2+} binding during DNA hydrolysis as revealed by anomalous signal of Mn^{2+}. The $2F_o$-F_c map for Me^{2+}, DNA, waters (red spheres), and catalytic residues (blue) was contoured at 2.0 σ. The anomalous map for Mn^{2+} was contoured at 3.0 σ. (**E**) Tl^{+} concentration on DNA cleavage. Precipitation was detected when Tl^{+} was at or greater than 150 mM. (**F**) Additional Na^{+} concentration on DNA cleavage. (**E, F**) The errors bars represent the standard deviation while the points represent the mean of triplicate measurements for cleaved DNA product.

bioRxiv preprint doi: https://doi.org/10.1101/2024.05.02.592236; this version posted May 5, 2024. The copyright holder for this preprint (which was not certified by peer review) is the author/funder, who has granted bioRxiv a license to display the preprint in perpetuity. It is made available under aCC-BY 4.0 International license.

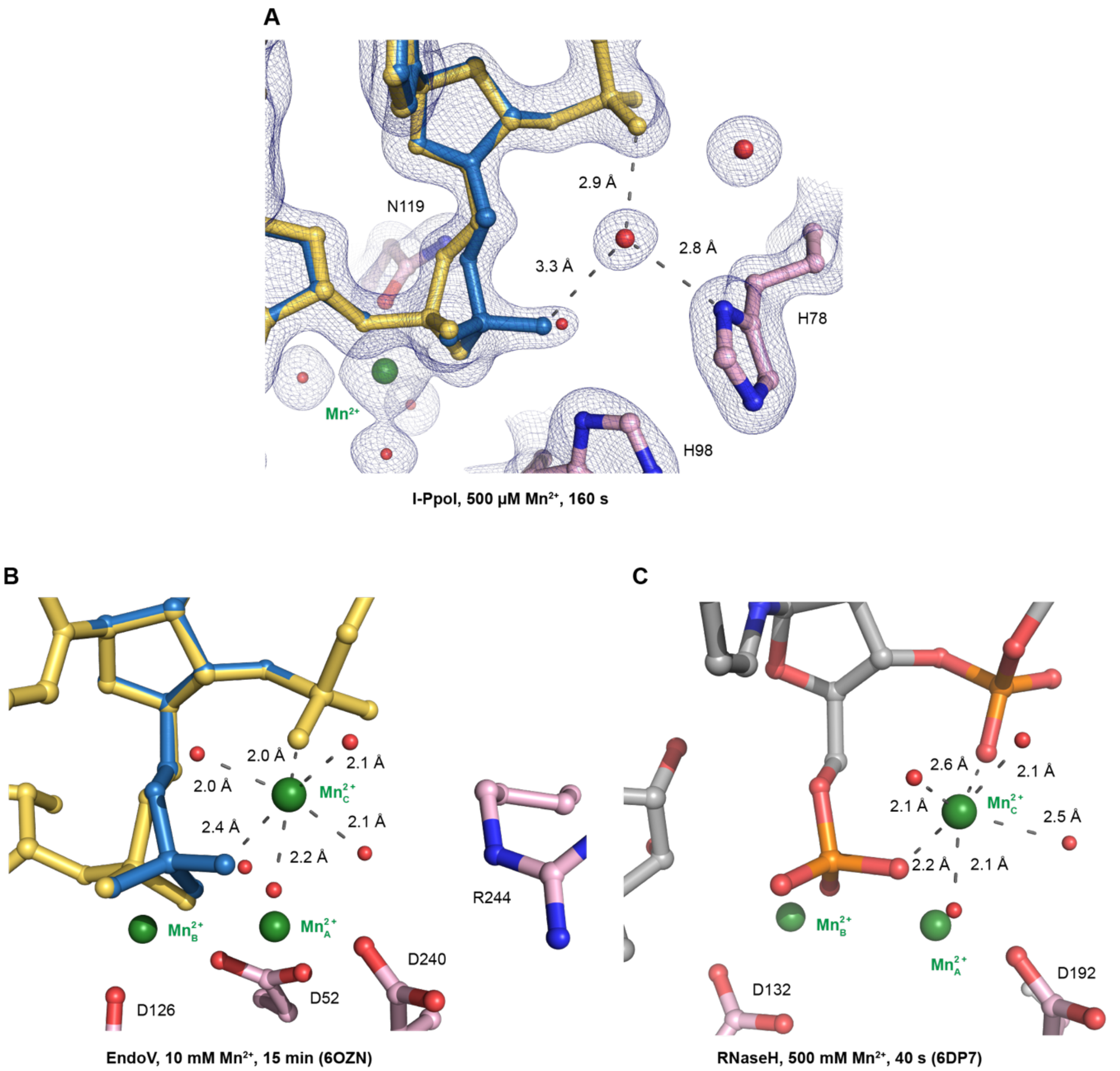

Fig. S5. Speculative ligand environment of the transient Me^{2+} in I-PpoI (A) in comparison to EndoV (B) and RNaseH (C).

(**A**) The $2F_o$-F_c map for Me^{2+}, DNA, waters (red spheres), and catalytic residues (blue) was contoured at 2.0 σ. The DNA phosphate conformation within the active site of I-PpoI is looser in comparison to that in EndoV and RNaseH to bind an additional Me^{2+}. Carbon atoms of residues within the active site are colored in pink. The Me^{2+} are depicted by green spheres while waters are depicted by red spheres. (**A, B**) the DNA reactant state is colored in yellow while the DNA product state is colored in blue.

bioRxiv preprint doi: https://doi.org/10.1101/2024.05.02.592236; this version posted May 5, 2024. The copyright holder for this preprint (which was not certified by peer review) is the author/funder, who has granted bioRxiv a license to display the preprint in perpetuity. It is made available under aCC-BY 4.0 International license.

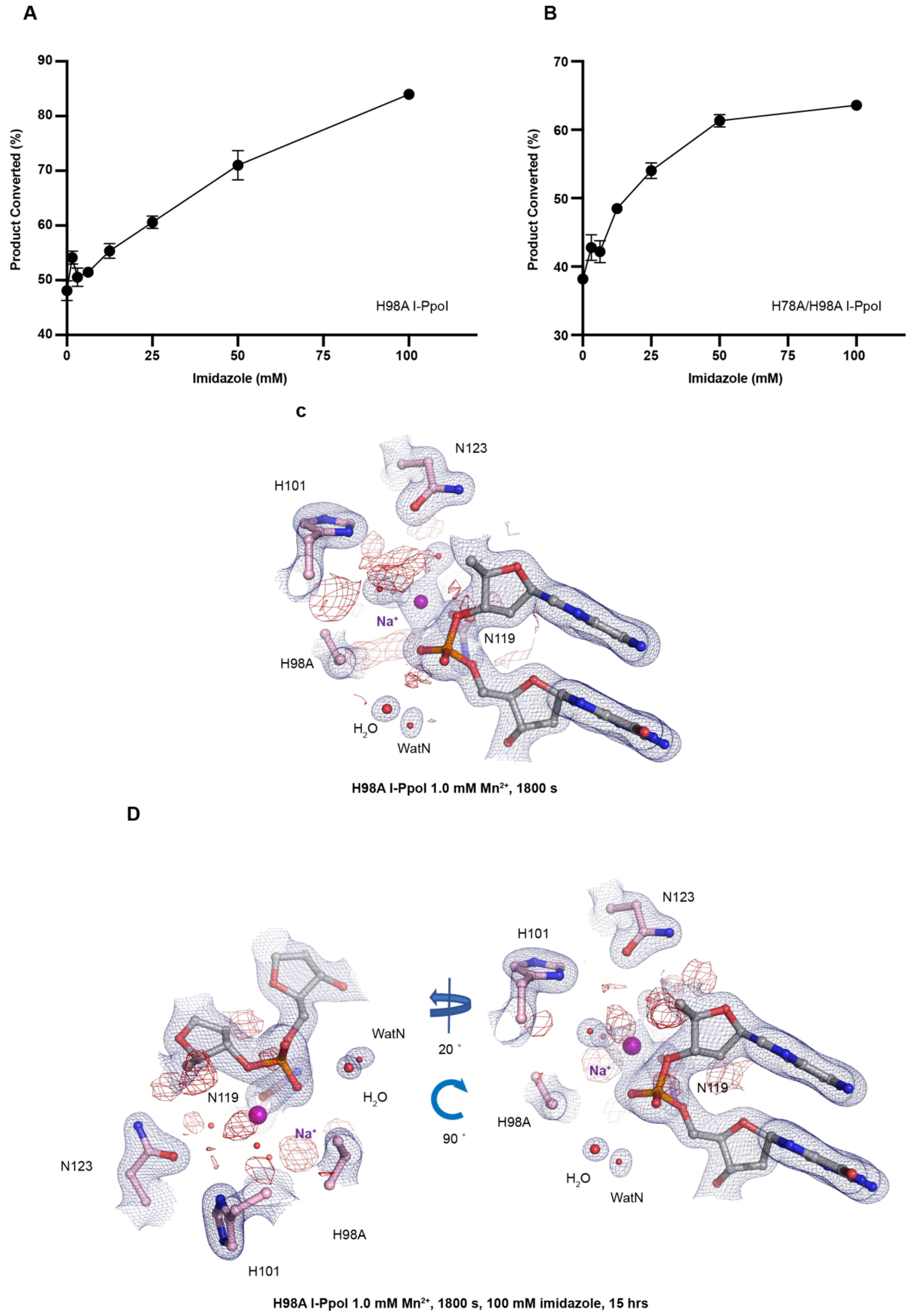

Fig. S6. Histidine I-PpoI mutants and partial rescued cleavage activity by imidazole.
(**A**) Titration of imidazole on DNA cleavage activity by H98A I-PpoI. (**B**) Titration of imidazole on DNA cleavage activity by H78A/H98A I-PpoI. (**A, B**) The errors bars represent the standard deviation while the points represent the mean of duplicate measurements for cleaved DNA product. (**C**) Structure of H98A I-PpoI active site after 1 mM Mn^{2+} soaking for 1800 s. (**D**) Structure of

bioRxiv preprint doi: https://doi.org/10.1101/2024.05.02.592236; this version posted May 5, 2024. The copyright holder for this preprint (which was not certified by peer review) is the author/funder, who has granted bioRxiv a license to display the preprint in perpetuity. It is made available under aCC-BY 4.0 International license.

H98A I-PpoI active site after 100 mM imidazole for 15 hrs following 1 mM Mn^{2+} soaking for 1800 s. The anomalous map for Mn^{2+} was contoured at 2.0 σ. (**A, D**) The $2F_o$-F_c map for Me^{2+}, DNA, waters (red spheres), and catalytic residues (blue) was contoured at 2.0 σ.

bioRxiv preprint doi: https://doi.org/10.1101/2024.05.02.592236; this version posted May 5, 2024. The copyright holder for this preprint (which was not certified by peer review) is the author/funder, who has granted bioRxiv a license to display the preprint in perpetuity. It is made available under aCC-BY 4.0 International license.

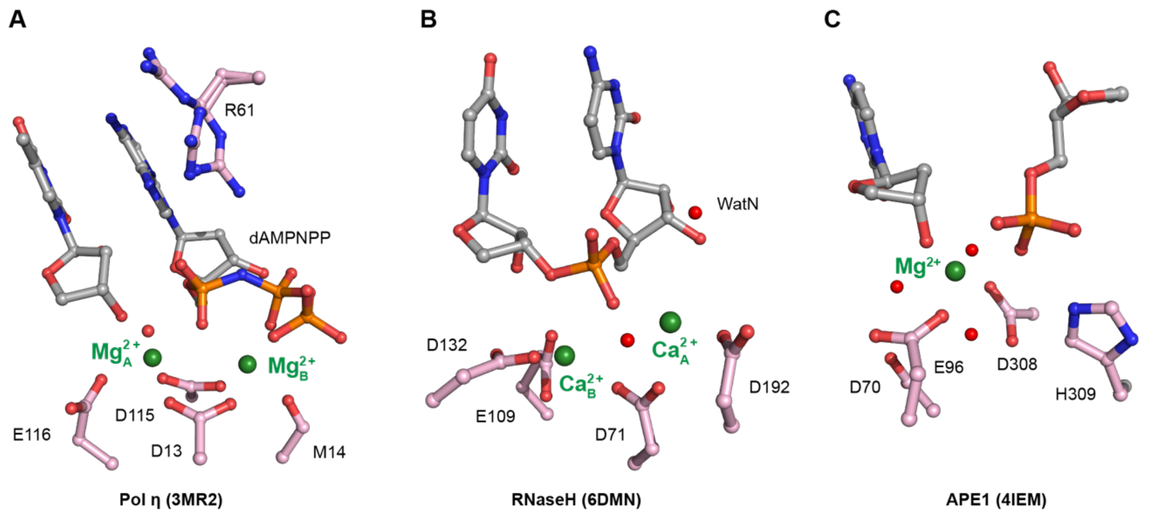

Fig. S7. Active sites of Pol η in (A), RNaseH in (B), and APE1 in (C).
Carbon atoms of residues within the active site are colored in pink. The Me^{2+} are depicted by green spheres while waters are depicted by red spheres.

bioRxiv preprint doi: https://doi.org/10.1101/2024.05.02.592236; this version posted May 5, 2024. The copyright holder for this preprint (which was not certified by peer review) is the author/funder, who has granted bioRxiv a license to display the preprint in perpetuity. It is made available under aCC-BY 4.0 International license.

Table S1. Crystal Diffraction and refinement data.

(**A**) I-PpoI-DNA complex reaction with 500 μM Mg^{2+} at pH 7.0.

	PRS (pH 7.0)	10 s	20 s	40 s
**PDB Code**	8VMO	8VMP	8VMQ	8VMR
**Data collection**				
Wavelength (Å)	0.9786	0.9787	0.9787	0.9787
Space group	*P3₁21*	*P3₁21*	*P3₁21*	*P3₁21*
Cell dimensions				
a, b, c (Å)	114.083	113.58	113.58	113.58
	114.083	113.58	113.58	113.58
	88.123	87.99	87.99	87.99
α, β, γ (˚)	90, 90, 120	90, 90, 120	90, 90, 120	90, 90, 120
Resolution (Å)[1]	49.4 - 1.68	37.18 - 1.45	37.18 - 1.48	37.18 - 1.5
	(1.74 - 1.68)	(1.502 - 1.45)	(1.533 - 1.48)	(1.554 - 1.5)
R_{sym} or R_{merge}[1]	0.09259 (0.9459)	0.08503 (0.7265)	0.07876 (0.9328)	0.08952 (1.033)
$I/\sigma I$[1]	15.60 (2.31)	13.95 (2.24)	16.47 (2.20)	14.32 (1.95)
$CC^{1/2}$ [1]	0.999 (0.838)	0.998 (0.836)	0.999 (0.803)	0.999 (0.814)
Completeness (%)	99.99 (100.00)	99.50 (98.81)	99.90 (99.21)	99.97 (99.86)
Redundancy[1]	11.0 (10.9)	9.0 (8.3)	10.9 (10.1)	10.9 (10.2)
No. unique reflections[1]	75539 (7463)	115178 (11274)	108855 (10719)	104681 (10370)
**Refinement**				
Me_1	1.0 Na^+	1.0 Na^+	0.1 Mg^{2+}	0.2 Mg^{2+}
			0.9 Na^+	0.8 Na^+
Me_2	1.0 Na^+	1.0 Na^+	1.0 Na^+	1.0 Na^+
$Product_1$	-	-	0.1	0.2
$Product_2$	-	-	-	-
B-factors				
Me_1/Lig_1 [2]	20.7/22.0	15.3/16.6	15.9/17.5	15.4/17.2
Me_2/Lig_2 [2]	16.7/21.0	15.3/16.3	15.2/17.0	15.5/17.5
Protein	24.69	19.91	20.18	20.6
DNA	27.55	22.71	22.55	23.27
Ligand	19.88	15.38	15.64	15.79
Water	31.07	25.52	26.83	26.55
Resolution (Å)	1.68	1.45	1.48	1.5
No. reflections	75534 (7463)	115174 (11274)	108850 (10719)	104674 (10370)
R_{work}/R_{free}	0.17/0.19	0.18/0.19	0.17/0.19	0.18/0.19
Wilson B	22.59	17.58	17.71	17.79
Ramachandran				
Favored (%)	99.06	99.69	99.38	100
Outlier (%)	0	0	0	0
R.m.s. deviations				
Bond lengths (Å)	0.009	0.008	0.01	0.008
Bond angles (˚)	1.08	1.07	1.19	1.09

[1]Data in the highest resolution shell is shown in the parenthesis.

[2]B-factor of metal ions and their protein nucleotide ligands.

bioRxiv preprint doi: https://doi.org/10.1101/2024.05.02.592236; this version posted May 5, 2024. The copyright holder for this preprint (which was not certified by peer review) is the author/funder, who has granted bioRxiv a license to display the preprint in perpetuity. It is made available under aCC-BY 4.0 International license.

	80 s	160 s	320 s	600 s
**PDB Code**	8VMS	8VMT	8VMU	8VMV
**Data collection**				
Wavelength (Å)	0.9787	0.9787	0.9787	0.9787
Space group	$P3_121$	$P3_121$	$P3_121$	$P3_121$
Cell dimensions				
a, b, c (Å)	113.58	113.58	113.58	113.58
	113.58	113.58	113.58	113.58
	87.99	87.99	87.99	87.99
α, β, γ (°)	90, 90, 120	90, 90, 120	90, 90, 120	90, 90, 120
Resolution (Å)[1]	37.18 - 1.42	42.93 - 1.481	37.18 - 1.52	37.18 - 1.59
	(1.471 - 1.42)	(1.534 - 1.481)	(1.574 - 1.52)	(1.574 - 1.52)
R_{sym} or R_{merge}[1]	0.09766 (0.8326)	0.07917 (0.9958)	0.08757 (0.8598)	0.1071 (0.8005)
$I/\sigma I$[1]	12.89 (1.95)	17.59 (2.49)	14.07 (2.11)	12.43 (2.24)
$CC^{1/2}$[1]	0.998 (0.82)	0.999 (0.811)	0.999 (0.851)	0.998 (0.869)
Completeness (%)	99.89 (99.08)	99.98 (99.99)	99.91 (99.37)	99.97 (100.00)
Redundancy[1]	10.8 (9.9)	11.0 (10.6)	10.9 (10.7)	11.0 (11.1)
No. unique reflections[1]	123050 (12056)	108688 (10781)	100572 (9925)	88017 (8714)
**Refinement**				
Me_1	0.25 Mg^{2+}	0.5 Mg^{2+}	0.7 Mg^{2+}	1.0 Mg^{2+}
	0.75 Na^+	0.5 Na^+	0.3 Na^+	
Me_2	0.2 Mg^{2+}	0.4 Mg^{2+}	0.6 Mg^{2+}	0.9 Mg^{2+}
	0.8 Na^+	0.6 Na^+	0.4 Na^+	0.1 Na^+
$Product_1$	0.25	0.5	0.7	1.0
$Product_2$	0.2	0.4	0.6	0.9
B-factors				
Me_1/Lig_1[2]	15.4/17.8	15.1/18.0	15.0/17.6	13.8/15.9
Me_2/Lig_2[2]	15.8/17.5	15.8/17.5	16.3/17.4	14.4/15.7
Protein	20.05	20.07	20.42	19.41
DNA	22.25	22.28	23.31	22.02
Ligand	15.54	15.38	15.93	14.57
Water	26.41	26.15	26.35	24.89
Resolution (Å)	1.42	1.48	1.52	1.52
No. reflections	123047 (12056)	108678 (10781)	100559 (9925)	88005 (8714)
R_{work}/R_{free}	0.18/0.19	0.18/0.20	0.18/0.19	0.18/0.20
Wilson B	17.52	17.17	17.21	16.85
Ramachandran				
Favored (%)	99.69	99.69	99.38	99.69
Outlier (%)	0	0	0	0
R.m.s. deviations				
Bond lengths (Å)	0.008	0.009	0.009	0.022
Bond angles (°)	1.06	1.1	1.12	1.55

[1]Data in the highest resolution shell is shown in the parenthesis.

[2]B-factor of metal ions and their protein nucleotide ligands.

bioRxiv preprint doi: https://doi.org/10.1101/2024.05.02.592236; this version posted May 5, 2024. The copyright holder for this preprint (which was not certified by peer review) is the author/funder, who has granted bioRxiv a license to display the preprint in perpetuity. It is made available under aCC-BY 4.0 International license.

(**B**) I-PpoI-DNA complex reaction with 500 μM Mg^{2+} at pH 6.0.

ph6	PRS (pH 6.0)	10 s	20 s
**PDB Code**	8VMW	8VMX	8VMY
**Data collection**			
Wavelength (Å)	0.9765	0.9765	0.9765
Space group	$P3_121$	$P3_121$	$P3_121$
Cell dimensions			
a, b, c (Å)	113.88	113.88	113.88
	113.88	113.88	113.88
	88.22	88.22	88.22
α, β, γ (˚)	90, 90, 120	90, 90, 120	90, 90, 120
Resolution (Å)[1]	47.84 - 1.6	47.84 - 1.45	43.04 - 1.53
	(1.657 - 1.6)	(1.502 - 1.45)	(1.585 - 1.53)
R_{sym} or R_{merge}[1]	0.06892 (0.8392)	0.06899 (0.8012)	0.08801 (0.7433)
$I/\sigma I$[1]	21.67 (2.41)	20.39 (2.42)	14.38 (1.81)
$CC^{1/2}$[1]	0.999 (0.829)	0.999 (0.826)	0.999 (0.845)
Completeness (%)	99.88 (99.99)	99.95 (99.66)	99.74 (97.54)
Redundancy[1]	9.9 (9.6)	9.9 (9.3)	9.7 (9.2)
No. unique reflections[1]	86963 (8596)	116644 (11543)	99254 (9632)
**Refinement**			
Me_1	1.0 Na^+	1.0 Na^+	0.05 Mg^{2+}
			0.95 Na^+
Me_2	1.0 Na^+	1.0 Na^+	1.0 Na^+
$Product_1$	-	-	0.05
$Product_2$	-	-	-
B-factors			
Me_1/Lig_1[2]	14.5/16.6	13.1/14.7	13.0/13.9
Me_2/Lig_2[2]	115.0/15.7	13.1/14.2	13.2/14.8
Protein	18.25	17.45	17.75
DNA	21.07	20.15	20.36
Ligand	14.37	13.05	13.2
Water	24.16	23.17	23.99
Resolution (Å)	1.6	1.45	1.53
No. reflections	86958 (8596)	116640 (11543)	99251 (9632)
R_{work}/R_{free}	0.18/0.20	0.18/0.18	0.18/0.19
Wilson B	15.76	15.32	15.54
Ramachandran			
Favored (%)	99.06	98.75	99.06
Outlier (%)	0	0	0
R.m.s. deviations			
Bond lengths (Å)	0.008	0.008	0.01
Bond angles (˚)	1.11	1.1	1.16

[1]Data in the highest resolution shell is shown in the parenthesis.

[2]B-factor of metal ions and their protein nucleotide ligands.

bioRxiv preprint doi: https://doi.org/10.1101/2024.05.02.592236; this version posted May 5, 2024. The copyright holder for this preprint (which was not certified by peer review) is the author/funder, who has granted bioRxiv a license to display the preprint in perpetuity. It is made available under aCC-BY 4.0 International license.

	40 s	80 s	160 s
**PDB Code**	8VMZ	8VN0	8VN1
**Data collection**			
Wavelength (Å)	0.9765	0.9765	0.9765
Space group	*P3₁21* → $P3_121$	$P3_121$	$P3_121$
Cell dimensions			
a, b, c (Å)	113.88	113.88	113.88
	113.88	113.88	113.88
	88.22	88.22	88.22
α, β, γ (˚)	90, 90, 120	90, 90, 120	90, 90, 120
Resolution (Å)[1]	43.04 - 1.57	43.04 - 1.6	43.04 - 1.79
	(1.626 - 1.57)	(1.657 - 1.6)	(1.854 - 1.79)
R_{sym} or R_{merge}[1]	0.06753 (0.8942)	0.1128 (0.8972)	0.1035 (0.8058)
$I/\sigma I$[1]	22.77 (2.47)	12.65 (2.74)	16.88 (2.64)
$CC^{1/2}$[1]	1 (0.824)	0.997 (0.816)	0.999 (0.849)
Completeness (%)	99.64 (96.64)	99.96 (99.98)	99.88 (99.48)
Redundancy[1]	10.0 (9.6)	9.9 (9.7)	9.9 (8.9)
No. unique reflections[1]	91821 (8875)	87042 (8595)	62370 (6119)
**Refinement**			
Me_1	0.1 Mg^{2+}	0.2 Mg^{2+}	0.3 Mg^{2+}
	0.9 Na^{+}	0.8 Na^{+}	0.7 Na^{+}
Me_2	0.1 Mg^{2+}	0.2 Mg^{2+}	0.3 Mg^{2+}
	0.9 Na^{+}	0.8 Na^{+}	0.7 Na^{+}
$Product_1$	0.1	0.2	0.3
$Product_2$	0.1	0.2	0.3
B-factors			
Me_1/Lig_1[2]	12.9/14.8	13.0/14.9	14.7/16.6
Me_2/Lig_2[2]	13.0/14.5	12.9/14.0	14.2/15.1
Protein	17.67	17.32	17.42
DNA	19.82	19.28	20.43
Ligand	13.12	12.76	14
Water	23.98	24.15	23.84
Resolution (Å)	1.57	1.6	1.79
No. reflections	91816 (8875)	87034 (8595)	62365 (6118)
R_{work}/R_{free}	0.18/0.20	0.18/0.20	0.18/0.20
Wilson B	14.96	14.21	16.34
Ramachandran			
Favored (%)	99.38	99.38	99.38
Outlier (%)	0	0	0
R.m.s. deviations			
Bond lengths (Å)	0.008	0.009	0.008
Bond angles (˚)	1.14	1.13	1.09

[1]Data in the highest resolution shell is shown in the parenthesis.
[2]B-factor of metal ions and their protein nucleotide ligands.

bioRxiv preprint doi: https://doi.org/10.1101/2024.05.02.592236; this version posted May 5, 2024. The copyright holder for this preprint (which was not certified by peer review) is the author/funder, who has granted bioRxiv a license to display the preprint in perpetuity. It is made available under aCC-BY 4.0 International license.

	320 s	600 s	1200 s
**PDB Code**	8VN2	8VN3	8VN4
**Data collection**			
Wavelength (Å)	0.9765	0.9765	0.9765
Space group	$P3_121$	$P3_121$	$P3_121$
Cell dimensions			
a, b, c (Å)	113.88	113.88	113.88
	113.88	113.88	113.88
	88.22	88.22	88.22
α, β, γ (˚)	90, 90, 120	90, 90, 120	90, 90, 120
Resolution (Å)[1]	43.04 - 1.63	43.04 - 1.63	43.04 - 1.75
	(1.688 - 1.63)	(1.688 - 1.63)	(1.813 - 1.75)
R_{sym} or R_{merge}[1]	0.09884 (0.8855)	0.08108 (0.8274)	0.1042 (0.8633)
$I/\sigma I$[1]	13.94 (1.90)	19.75 (2.62)	13.09 (1.81)
$CC^{1/2}$ [1]	0.999 (0.815)	0.999 (0.844)	0.998 (0.843)
Completeness (%)	99.87 (99.42)	99.98 (99.99)	99.93 (99.73)
Redundancy[1]	9.8 (9.7)	10.0 (10.2)	9.7 (9.8)
No. unique reflections[1]	82333 (8100)	82420 (8146)	66724 (6607)
**Refinement**			
Me_1	0.6 Mg^{2+}	0.7 Mg^{2+}	0.95 Mg^{2+}
	0.4 Na^{+}	0.3 Na^{+}	0.05 Na^{+}
Me_2	0.4 Mg^{2+}	0.65 Mg^{2+}	0.95 Mg^{2+}
	0.6 Na^{+}	0.35 Na^{+}	0.05 Na^{+}
$Product_1$	0.6	0.65/0.05	0.80/0.15
$Product_2$	0.4	0.6/0.05	0.70/0.25
B-factors			
Me_1/Lig_1 [2]	14.3/16.0	13.9/16.2	13.5/15.2
Me_2/Lig_2 [2]	14.7/15.0	13.9/15.8	12.6/15.4
Protein	17.93	17.52	18.08
DNA	20.32	20.1	20.75
Ligand	13.85	13.49	13.19
Water	25.15	24.55	24.66
Resolution (Å)	1.63	1.63	1.75
No. reflections	82327 (8099)	82412 (8145)	66716 (6607)
R_{work}/R_{free}	0.18/0.19	0.18/0.19	0.18/0.20
Wilson B	14.97	14.9	15.67
Ramachandran			
Favored (%)	99.38	99.38	99.38
Outlier (%)	0	0	0
R.m.s. deviations			
Bond lengths (Å)	0.008	0.009	0.009
Bond angles (˚)	1.17	1.16	1.13

[1]Data in the highest resolution shell is shown in the parenthesis.
[2]B-factor of metal ions and their protein nucleotide ligands.

bioRxiv preprint doi: https://doi.org/10.1101/2024.05.02.592236; this version posted May 5, 2024. The copyright holder for this preprint (which was not certified by peer review) is the author/funder, who has granted bioRxiv a license to display the preprint in perpetuity. It is made available under aCC-BY 4.0 International license.

(**C**) I-PpoI-DNA complex reaction with 500 µM Mg^{2+} at pH 8.0.

	PRS (pH 8.0)	10 s	20 s
**PDB Code**	8VN5	8VN6	8VN7
**Data collection**			
Wavelength (Å)	0.97872	0.9787	0.9787
Space group	*P3₁21*	*P3₁21*	*P3₁21*
Cell dimensions			
a, b, c (Å)	113.65<br>113.65<br>88.31	113.65<br>113.65<br>88.31	113.65<br>113.65<br>88.31
α, β, γ (˚)	90, 90, 120	90, 90, 120	90, 90, 120
Resolution (Å)[1]	47.79 - 1.653<br>(1.712 - 1.653)	42.99 - 1.541<br>(1.596 - 1.541)	42.99 - 1.67<br>(1.73 - 1.67)
R_{sym} or R_{merge}[1]	0.0808 (1.057)	0.07081 (0.9637)	0.08404 (1.07)
$I/\sigma I$[1]	17.76 (2.20)	20.06 (2.26)	16.93 (2.14)
$CC^{1/2}$[1]	0.999 (0.822)	0.999 (0.806)	0.999 (0.811)
Completeness (%)	99.69 (97.12)	99.70 (97.14)	99.58 (95.98)
Redundancy[1]	11.1 (10.8)	11.0 (10.8)	11.1 (10.6)
No. unique reflections[1]	78663 (7600)	96789 (9311)	76126 (7246)
**Refinement**			
Me_1	1.0 Na^+	0.15 Mg^{2+}<br>0.85 Na^+	0.35 Mg^{2+}<br>0.65 Na^+
Me_2	1.0 Na^+	0.15 Mg^{2+}<br>0.85 Na^+	0.25 Mg^{2+}<br>0.75 Na^+
$Product_1$	-	0.15	0.35
$Product_2$	-	0.15	0.25
B-factors			
Me_1/Lig_1[2]	17.3/19.3	16.7/18.6	16.3/18.4
Me_2/Lig_2[2]	17.1/18.4	16.9/18.3	16.6/18.4
Protein	21.73	21.68	21.36
DNA	24.16	23.65	23.57
Ligand	17.09	16.87	16.49
Water	28.9	28.62	28.61
Resolution (Å)	1.65	1.54	1.67
No. reflections	78651 (7597)	96776 (9311)	76108 (7241)
R_{work}/R_{free}	0.17/0.19	0.18/0.20	0.18/0.20
Wilson B	18.36	19.47	18.36
Ramachandran			
Favored (%)	99.38	99.06	99.69
Outlier (%)	0	0.31	0
R.m.s. deviations			
Bond lengths (Å)	0.008	0.008	0.009
Bond angles (˚)	1.12	1.1	1.11

[1]Data in the highest resolution shell is shown in the parenthesis.
[2]B-factor of metal ions and their protein nucleotide ligands.

bioRxiv preprint doi: https://doi.org/10.1101/2024.05.02.592236; this version posted May 5, 2024. The copyright holder for this preprint (which was not certified by peer review) is the author/funder, who has granted bioRxiv a license to display the preprint in perpetuity. It is made available under aCC-BY 4.0 International license.

	40 s	80 s	160 s
**PDB Code**	8VN8	8VN9	8VNA
**Data collection**			
Wavelength (Å)	0.9787	0.9787	0.9787
Space group	$P3_121$	$P3_121$	$P3_121$
Cell dimensions			
a, b, c (Å)	113.65	113.65	113.65
	113.65	113.65	113.65
	88.31	88.31	88.31
α, β, γ (˚)	90, 90, 120	90, 90, 120	90, 90, 120
Resolution (Å)[1]	47.79 - 1.6	42.99 - 1.69	34.87 - 1.543
	(1.657 - 1.6)	(1.75 - 1.69)	(1.598 - 1.543)
R_{sym} or R_{merge}[1]	0.08194 (1.012)	0.08947 (0.9124)	0.08164 (0.9551)
$I/\sigma I$[1]	17.51 (2.23)	15.59 (2.16)	16.71 (2.22)
$CC^{1/2}$[1]	0.999 (0.79)	0.999 (0.845)	0.999 (0.818)
Completeness (%)	99.74 (97.60)	99.72 (97.37)	99.98 (99.98)
Redundancy[1]	11.0 (10.8)	11.0 (10.5)	11.0 (11.0)
No. unique reflections[1]	86597 (8362)	73645 (7158)	96719 (9584)
**Refinement**			
Me_1	0.45 Mg^{2+}	0.65 Mg^{2+}	0.95 Mg^{2+}
	0.55 Na^+	0.35 Na^+	0.05 Na^+
Me_2	0.3 Mg^{2+}	0.5 Mg^{2+}	0.7 Mg^{2+}
	0.7 Na^+	0.5 Na^+	0.3 Na^+
$Product_1$	0.45	0.65	0.95
$Product_2$	0.3	0.5	0.7
B-factors			
Me_1/Lig_1[2]	16.8/19.4	16.7/19.0	16.3/18.3
Me_2/Lig_2[2]	17.3/18.5	17.7/18.9	16.7/18.6
Protein	22.2	22.85	22.03
DNA	24.43	25.14	24.45
Ligand	17.18	17.61	16.84
Water	29.42	29.72	29.13
Resolution (Å)	1.6	1.69	1.54
No. reflections	86585 (8362)	73632 (7154)	96712 (9584)
R_{work}/R_{free}	0.18/0.19	0.18/0.19	0.18/0.20
Wilson B	19.09	19.87	19.48
Ramachandran			
Favored (%)	99.69	100	99.69
Outlier (%)	0	0	0
R.m.s. deviations			
Bond lengths (Å)	0.009	0.009	0.009
Bond angles (˚)	1.15	1.13	1.17

[1]Data in the highest resolution shell is shown in the parenthesis.
[2]B-factor of metal ions and their protein nucleotide ligands.

bioRxiv preprint doi: https://doi.org/10.1101/2024.05.02.592236; this version posted May 5, 2024. The copyright holder for this preprint (which was not certified by peer review) is the author/funder, who has granted bioRxiv a license to display the preprint in perpetuity. It is made available under aCC-BY 4.0 International license.

	240 s	320 s	600 s
**PDB Code**	8VNB	8VNC	8VND
**Data collection**			
Wavelength (Å)	0.9787	0.9787	0.9787
Space group	*P3₁21* → $P3_121$	$P3_121$	$P3_121$
Cell dimensions			
a, b, c (Å)	113.65	113.65	113.65
	113.65	113.65	113.65
	88.31	88.31	88.31
α, β, γ (˚)	90, 90, 120	90, 90, 120	90, 90, 120
Resolution (Å)[1]	42.99 - 1.72	47.79 - 1.623	47.79 - 1.602
	(1.781 - 1.72)	(1.681 - 1.623)	(1.659 - 1.602)
R_{sym} or R_{merge}[1]	0.1043 (0.9116)	0.08664 (0.9454)	0.09777 (0.9793)
$I/\sigma I$[1]	14.41 (2.29)	16.52 (2.41)	14.55 (2.57)
$CC^{1/2}$[1]	0.999 (0.844)	0.999 (0.839)	0.998 (0.827)
Completeness (%)	99.95 (99.68)	99.98 (100.00)	99.98 (100.00)
Redundancy[1]	11.0 (10.9)	11.0 (11.1)	11.2 (11.1)
No. unique reflections[1]	70052 (6923)	83221 (8204)	86506 (8558)
**Refinement**			
Me_1	1.0 Mg^{2+}	1.0 Mg^{2+}	1.0 Mg^{2+}
Me_2	1.0 Mg^{2+}	1.0 Mg^{2+}	1.0 Mg^{2+}
$Product_1$	1.0	1.0	1.0
$Product_2$	1.0	1.0	1.0
B-factors			
Me_1/Lig_1[2]	15.3/16.0	14.3/15.4	14.3/14.9
Me_2/Lig_2[2]	15.2/16.2	14.1/15.6	13.4/15.2
Protein	21.5	21.23	20.45
DNA	24.62	24.06	23.37
Ligand	16.41	15.79	15.13
Water	27.43	27.6	26.89
Resolution (Å)	1.72	1.62	1.6
No. reflections	70046 (6922)	83212 (8204)	86496 (8558)
R_{work}/R_{free}	0.18/0.20	0.18/0.19	0.18/0.19
Wilson B	19.61	18.78	17.95
Ramachandran			
Favored (%)	100	99.69	99.69
Outlier (%)	0	0	0
R.m.s. deviations			
Bond lengths (Å)	0.009	0.009	0.008
Bond angles (˚)	1.16	1.16	1.1

[1]Data in the highest resolution shell is shown in the parenthesis.
[2]B-factor of metal ions and their protein nucleotide ligands.

bioRxiv preprint doi: https://doi.org/10.1101/2024.05.02.592236; this version posted May 5, 2024. The copyright holder for this preprint (which was not certified by peer review) is the author/funder, who has granted bioRxiv a license to display the preprint in perpetuity. It is made available under aCC-BY 4.0 International license.

(**D**) I-PpoI-DNA complex reaction with 500 µM Mn^{2+} at pH 6.0.

	10 s	20 s	40 s	80 s
**PDB Code**	8VNE	8VNF	8VNG	8VNH
**Data collection**				
Wavelength (Å)	0.9786	0.9786	0.9786	0.9786
Space group	$P3_121$	$P3_121$	$P3_121$	$P3_121$
Cell dimensions				
a, b, c (Å)	114.06<br>114.06<br>88.02	114.06<br>114.06<br>88.02	114.06<br>114.06<br>88.02	114.06<br>114.06<br>88.02
α, β, γ (˚)	90, 90, 120	90, 90, 120	90, 90, 120	90, 90, 120
Resolution (Å)[1]	32.93 - 1.57<br>(1.626 - 1.57)	37.33 - 1.5<br>(1.554 - 1.5)	34.37 - 1.6<br>(1.657 - 1.6)	49.39 - 1.76<br>(1.823 - 1.76)
R_{sym} or R_{merge}[1]	0.07871 (0.9045)	0.08083 (1.097)	0.07564 (0.9743)	0.1045 (1.001)
$I/\sigma I$[1]	18.11 (2.53)	16.89 (2.09)	19.55 (2.40)	13.57 (2.13)
$CC^{1/2}$[1]	0.999 (0.841)	0.999 (0.821)	0.999 (0.809)	0.998 (0.832)
Completeness (%)	99.98 (100.00)	99.99 (100.00)	99.97 (100.00)	99.70 (97.24)
Redundancy[1]	11.1 (11.1)	11.0 (11.0)	11.1 (11.0)	11.0 (10.9)
No. unique reflections[1]	92218 (9154)	105592 (10465)	87153 (8627)	65495 (6311)
**Refinement**				
Me_1	0.05 Mn^{2+}<br>0.95 Na^+	0.1 Mn^{2+}<br>0.9 Na^+	0.15 Mn^{2+}<br>0.85 Na^+	0.2 Mn^{2+}<br>0.8 Na^+
Me_2	0.05 Mn^{2+}<br>0.95 Na^+	0.1 Mn^{2+}<br>0.9 Na^+	0.15 Mn^{2+}<br>0.85 Na^+	0.2 Mn^{2+}<br>0.8 Na^+
$Product_1$	0.05	0.1	0.15	0.2
$Product_2$	0.05	0.1	0.15	0.2
B-factors				
Me_1/Lig_1[2]	15.6/18.4	16.4/18.5	16.2/18.7	14.3/17.8
Me_2/Lig_2[2]	15.9/17.2	16.8/17.7	16.3/17.4	15.0/16.6
Protein	20.95	21.18	21.5	21.18
DNA	23.45	23.56	23.85	23.64
Ligand	16.09	16.72	16.52	15.39
Water	27.18	27.29	28.05	27.3
Resolution (Å)	1.57	1.5	1.6	1.76
No. reflections	92213 (9154)	105589 (10465)	87145 (8627)	65484 (6311)
R_{work}/R_{free}	0.18/0.20	0.18/0.20	0.18/0.19	0.18/0.21
Wilson B	18.8	18.7	18.63	18.68
Ramachandran				
Favored (%)	99.38	99.06	99.06	99.38
Outlier (%)	0	0	0	0
R.m.s. deviations				
Bond lengths (Å)	0.01	0.01	0.009	0.009
Bond angles (˚)	1.17	1.23	1.14	1.13

[1]Data in the highest resolution shell is shown in the parenthesis.
[2]B-factor of metal ions and their protein nucleotide ligands.

bioRxiv preprint doi: https://doi.org/10.1101/2024.05.02.592236; this version posted May 5, 2024. The copyright holder for this preprint (which was not certified by peer review) is the author/funder, who has granted bioRxiv a license to display the preprint in perpetuity. It is made available under aCC-BY 4.0 International license.

	120 s	160 s	240 s	320 s
**PDB Code**	8VNJ	8VNK	8VNL	8VNM
**Data collection**				
Wavelength (Å)	0.9786	0.9786	0.9786	0.9786
Space group	$P3_121$	$P3_121$	$P3_121$	$P3_121$
Cell dimensions				
a, b, c (Å)	114.06	114.06	114.06	114.06
	114.06	114.06	114.06	114.06
	88.02	88.02	88.02	88.02
α, β, γ (˚)	90, 90, 120	90, 90, 120	90, 90, 120	90, 90, 120
Resolution (Å)[1]	37.33 - 1.61	34.37 - 1.61	34.37 - 1.64	34.37 - 1.59
	(1.668 - 1.61)	(1.668 - 1.61)	(1.699 - 1.64)	(1.647 - 1.59)
R_{sym} or R_{merge}[1]	0.09422 (0.8953)	0.08262 (1.086)	0.07715 (0.814)	0.06716 (0.9081)
$I/\sigma I$[1]	15.78 (2.26)	21.04 (2.30)	17.91 (2.66)	22.99 (2.63)
$CC^{1/2}$[1]	0.999 (0.812)	0.999 (0.8)	0.999 (0.825)	1 (0.829)
Completeness (%)	99.98 (100.00)	99.97 (100.00)	99.30 (99.98)	99.97 (99.99)
Redundancy[1]	11.0 (11.1)	11.1 (11.1)	9.1 (9.0)	11.2 (11.1)
No. unique reflections[1]	85576 (8465)	85577 (8465)	80455 (8029)	88820 (8795)
**Refinement**				
Me_1	0.25 Mn^{2+}	0.4 Mn^{2+}	0.6 Mn^{2+}	0.8 Mn^{2+}
	0.75 Na^{+}	0.6 Na^{+}	0.4 Na^{+}	0.2 Na^{+}
Me_2	0.25 Mn^{2+}	0.45 Mn^{2+}	0.65 Mn^{2+}	0.8 Mn^{2+}
	0.75 Na^{+}	0.55 Na^{+}	0.35 Na^{+}	0.2 Na^{+}
$Product_1$	0.25	0.4	0.6	0.8
$Product_2$	0.25	0.45	0.65	0.8
B-factors				
Me_1/Lig_1[2]	16.8/19.1	15.8/18.9	15.2/17.0	15.6/17.0
Me_2/Lig_2[2]	16.6/17.7	15.5/17.9	14.4/17.1	14.3/16.4
Protein	21.3	21.4	20.27	21.02
DNA	24.16	24.16	23.05	23.84
Ligand	16.75	16.34	15.37	15.48
Water	27.58	27.91	26.79	27.97
Resolution (Å)	1.61	1.61	1.64	1.59
No. reflections	85571 (8465)	85566 (8465)	80444 (8028)	88812 (8794)
R_{work}/R_{free}	0.18/0.20	0.18/0.19	0.18/0.19	0.17/0.19
Wilson B	19.46	18.85	17.28	18.12
Ramachandran				
Favored (%)	99.38	99.38	100	100
Outlier (%)	0	0	0	0
R.m.s. deviations				
Bond lengths (Å)	0.009	0.009	0.008	0.008
Bond angles (˚)	1.17	1.13	1.04	1.07

[1]Data in the highest resolution shell is shown in the parenthesis.
[2]B-factor of metal ions and their protein nucleotide ligands.

bioRxiv preprint doi: https://doi.org/10.1101/2024.05.02.592236; this version posted May 5, 2024. The copyright holder for this preprint (which was not certified by peer review) is the author/funder, who has granted bioRxiv a license to display the preprint in perpetuity. It is made available under aCC-BY 4.0 International license.

	480 s	600 s
**PDB Code**	8VNN	8VNO
**Data collection**		
Wavelength (Å)	0.9786	0.9786
Space group	*P3₁21*	*P3₁21*
Cell dimensions		
a, b, c (Å)	114.06	114.06
	114.06	114.06
	88.02	88.02
α, β, γ (˚)	90, 90, 120	90, 90, 120
Resolution (Å)[1]	37.33 - 1.792	43.07 - 1.7
	(1.856 - 1.792)	(1.761 - 1.7)
R_{sym} or R_{merge}[1]	0.1312 (1.102)	0.08224 (0.9698)
$I/\sigma I$[1]	13.34 (2.57)	19.05 (2.56)
$CC^{1/2}$ [1]	0.998 (0.831)	0.999 (0.849)
Completeness (%)	99.97 (99.98)	99.97 (99.97)
Redundancy[1]	11.1 (11.1)	11.1 (11.2)
No. unique reflections[1]	62269 (6153)	72812 (7213)
**Refinement**		
Me_1	0.8 Mn^{2+}	0.9 Mn^{2+}
	0.2 Na^{+}	0.1 Na^{+}
Me_2	0.8 Mn^{2+}	0.9 Mn^{2+}
	0.2 Na^{+}	0.1 Na^{+}
$Product_1$	0.8	0.9
$Product_2$	0.8	0.9
B-factors		
Me_1/Lig_1 [2]	16.3/17.1	16.2/17.2
Me_2/Lig_2 [2]	14.7/16.7	15.5/17.2
Protein	22.06	21.53
DNA	25.03	24.79
Ligand	16.66	16.45
Water	28.18	27.81
Resolution (Å)	1.79	1.7
No. reflections	62260 (6152)	72799 (7211)
R_{work}/R_{free}	0.18/0.19	0.18/0.19
Wilson B	19.72	19.27
Ramachandran		
Favored (%)	100	99.69
Outlier (%)	0	0
R.m.s. deviations		
Bond lengths (Å)	0.01	0.009
Bond angles (˚)	1.22	1.21

[1]Data in the highest resolution shell is shown in the parenthesis.
[2]B-factor of metal ions and their protein nucleotide ligands.

bioRxiv preprint doi: https://doi.org/10.1101/2024.05.02.592236; this version posted May 5, 2024. The copyright holder for this preprint (which was not certified by peer review) is the author/funder, who has granted bioRxiv a license to display the preprint in perpetuity. It is made available under aCC-BY 4.0 International license.

(**E**) Other I-PpoI-DNA complexes.

	I-PpoI 0.2 M sodium malonate	His98Ala I-PpoI 1 mM Mn^{2+} 600 s	His98Ala I-PpoI 1 mM Mn^{2+} 15 h	200 mM Mn^{2+} 600 s
**PDB Code**	8VNP	8VNQ	8VNR	8VNS
**Data collection**				
Wavelength (Å)	1.1272	0.9765	0.9765	0.9765
Space group	*P3₁21*	*P3₁21*	*P3₁21*	*P3₁21*
Cell dimensions				
a, b, c (Å)	113.695	114.646	114.09	117.905
	113.695	114.646	114.09	117.905
	87.994	89.057	88.412	84.743
α, β, γ (˚)	90, 90, 120	90, 90, 120	90, 90, 120	90, 90, 120
Resolution (Å)[1]	34.28 - 1.79	43.36 - 1.93	43.13 - 1.98	43.73 - 2.111
	(1.854 - 1.79)	(1.999 - 1.93)	(2.051 - 1.98)	(2.186 - 2.111)
R_{sym} or R_{merge}[1]	0.05373 (0.1686)	0.21 (0.782)	0.1138 (0.8958)	0.1301 (0.6187)
$I/\sigma I$[1]	46.56 (15.80)	6.69 (1.37)	11.19 (1.62)	10.97 (2.08)
$CC^{1/2}$[1]	1 (0.995)	0.989 (0.848)	0.998 (0.848)	0.996 (0.905)
Completeness (%)	99.91 (99.95)	99.50 (95.26)	99.74 (98.19)	99.61 (96.97)
Redundancy[1]	20.5 (20.0)	9.8 (8.9)	9.8 (9.4)	9.7 (9.5)
No. unique reflections[1]	62043 (6149)	50811 (4824)	46448 (4491)	39247 (3780)
**Refinement**				
Me_1	1.0 Na	1.0 Na	1.0 Na	0.7 Mn^{2+}
				0.3 Na^{+}
Me_2	1.0 Na	1.0 Na	1.0 Na	0.8 Mn^{2+}
				0.2 Na^{+}
$Product_1$	-	-	-	0.7
$Product_2$	-	-	-	0.8
B-factors				
Me_1/Lig_1[2]	12.8/14.6	25.1/24.6	35.9/33.1	27.3/30.5
Me_2/Lig_2[2]	12.7/13.2	22.3/22.3	34.6/32.5	29.0/29.7
Protein	16.91	29.29	36.05	36.11
DNA	20.32	32.76	39.19	42.08
Ligand	13.7	23.81	33.27	29.82
Water	22.25	32.52	39.31	33.85
Resolution (Å)	1.79	1.93	1.98	2.11
No. reflections	62036 (6149)	50810 (4824)	46440 (4491)	39239 (3780)
R_{work}/R_{free}	0.17/0.19	0.19/0.21	0.19/0.22	0.22/0.25
Wilson B	16.51	30.06	35.07	35.57
Ramachandran				
Favored (%)	99.06	99.38	99.38	99.38
Outlier (%)	0	0	0	0
R.m.s. deviations				
Bond lengths (Å)	0.007	0.01	0.009	0.01
Bond angles (˚)	0.96	1.13	1.08	1.14

[1]Data in the highest resolution shell is shown in the parenthesis.
[2]B-factor of metal ions and their protein nucleotide ligands.

bioRxiv preprint doi: https://doi.org/10.1101/2024.05.02.592236; this version posted May 5, 2024. The copyright holder for this preprint (which was not certified by peer review) is the author/funder, who has granted bioRxiv a license to display the preprint in perpetuity. It is made available under aCC-BY 4.0 International license.

	500 µM Mg^{2+} 1800 s	70 mM Tl^{+} 1800 s
**PDB Code**	8VNT	8VNU
**Data collection**		
Wavelength (Å)	1.1271	0.9765
Space group	*P3₁21* → $P3_121$	$P3_121$
Cell dimensions		
a, b, c (Å)	114.129	113.574
	114.129	113.574
	88.74	87.695
α, β, γ (˚)	90, 90, 120	90, 90, 120
Resolution (Å)[1]	33.02 - 1.62	47.67 - 2.2
	(1.678 - 1.62)	(2.279 - 2.2)
R_{sym} or R_{merge}[1]	0.1009 (1.138)	0.1265 (0.9511)
$I/\sigma I$[1]	23.30 (2.78)	12.59 (2.17)
$CC^{1/2}$[1]	0.999 (0.867)	0.998 (0.837)
Completeness (%)	99.96 (99.96)	99.91 (100.00)
Redundancy[1]	20.2 (20.6)	9.9 (10.0)
No. unique reflections[1]	84795 (8426)	33498 (3304)
**Refinement**		
Me_1	1.0 Mg^{2+}	0.2 Tl^{+} 0.9 Na^{+}
Me_2	1.0 Mg^{2+}	0.2 Tl^{+} 0.8 Na^{+}
$Product_1$	0.75/0.25	0
$Product_2$	0.60/0.40	0
B-factors		
Me_1/Lig_1[2]	19.2/21.8	80/37.4
Me_2/Lig_2[2]	20.5/22.8	73.7/36.0
Protein	23.32	35.3
DNA	26.33	37.63
Ligand	19.65	59.35
Water	30.34	39.55
Resolution (Å)	1.62	2.2
No. reflections	84783 (8426)	33486 (3306)
R_{work}/R_{free}	0.18/0.20	0.18/0.22
Wilson B	21.49	36.08
Ramachandran		
Favored (%)	98.75	98.75
Outlier (%)	0	0.31
R.m.s. deviations		
Bond lengths (Å)	0.008	0.009
Bond angles (˚)	1.06	1.04

[1]Data in the highest resolution shell is shown in the parenthesis.
[2]B-factor of metal ions and their protein nucleotide ligands.
